# Evasion of the Cell-Mediated Immune Response by Alphaherpesviruses

**DOI:** 10.3390/v12121354

**Published:** 2020-11-26

**Authors:** Naoto Koyanagi, Yasushi Kawaguchi

**Affiliations:** 1 Division of Molecular Virology, Department of Microbiology and Immunology, the Institute of Medical Science, The University of Tokyo, Minato-ku, Tokyo 108-8639, Japan; 2 Department of Infectious Disease Control, International Research Center for Infectious Diseases, the Institute of Medical Science, The University of Tokyo, Minato-ku, Tokyo 108-8639, Japan; 3 Research Center for Asian Infectious Diseases, the Institute of Medical Science, The University of Tokyo, Minato-ku, Tokyo 108-8639, Japan

**Keywords:** alphaherpesvirus, adaptive immune response, herpesviral evasion

## Abstract

Alphaherpesviruses cause various diseases and establish life-long latent infections in humans and animals. These viruses encode multiple viral proteins and miRNAs to evade the host immune response, including both innate and adaptive immunity. Alphaherpesviruses evolved highly advanced immune evasion strategies to be able to replicate efficiently in vivo and produce latent infections with recurrent outbreaks. This review describes the immune evasion strategies of alphaherpesviruses, especially against cytotoxic host immune responses. Considering these strategies, it is important to evaluate whether the immune evasion mechanisms in cell cultures are applicable to viral propagation and pathogenicity in vivo. This review focuses on cytotoxic T lymphocytes (CTLs), natural killer cells (NK cells), and natural killer T cells (NKT cells), which are representative immune cells that directly damage virus-infected cells. Since these immune cells recognize the ligands expressed on their target cells via specific activating and/or inhibitory receptors, alphaherpesviruses make several ligands that may be targets for immune evasion. In addition, alphaherpesviruses suppress the infiltration of CTLs by downregulating the expression of chemokines at infection sites in vivo. Elucidation of the alphaherpesvirus immune evasion mechanisms is essential for the development of new antiviral therapies and vaccines.

## 1. Introduction

Herpesviridae is a large family of DNA viruses that cause various diseases in humans and animals. The Herpesviridae family is subdivided into the alpha-, beta-, and gammaherpesvirus subfamilies based on molecular and biological properties. Herpes simplex virus type 1 (HSV-1), HSV-2, and varicella-zoster virus (VZV) belong to the alphaherpesvirus subfamily. HSV-1 and HSV-2 include the etiologic agents of human oral herpes, genital herpes, herpes keratitis, neonatal herpes, and herpes encephalitis [1]. In human primary infections, HSV infects and proliferates in mucosal epithelial cells, attaches to sensory nerve endings, and then establishes a life-long latent infection in the trigeminal ganglia and/or dorsal root ganglia. Recurrent infections can arise by reactivation from the latent state by a variety of stimuli (e.g., physical and emotional stress, fever, exposure to ultraviolet light, and immune suppression) and cause pathology in humans [1]. Thus, HSV persists in its host with recurrent latent and lytic infections [1]. VZV primary infection leads to acute varicella (chickenpox), and then establishes a life-long latent infection in cranial nerves and dorsal root ganglia. VZV can reactivate as herpes zoster (HZ) or “shingles” [1]. The animal pseudorabies virus (PRV), equine herpesvirus 1 and 4 (EHV-1 and EHV-4), and bovine herpes virus 1 (BHV-1) also belong to the alphaherpesvirus subfamily. PRV is a porcine alphaherpesvirus and causes fever, loss of appetite, and miscarriage in pigs [2]. EHV-1 and EHV-4 are equine alphaherpesviruses and mainly cause fever, respiratory infections, miscarriages, and neurological symptoms in horses [3]. Marek’s disease virus (MDV) is a lymphotropic alphaherpesvirus and causes tumors of T lymphocytes in chickens [4]. Herpesviruses are highly host specific, but sometimes can be transmitted across species and produce a pathological condition leading to death [5,6]. Since herpesvirus infections are life-long, the virus must evade its host’s immune responses during latent infection to prevent it from being eliminated by its host [1]. Furthermore, even in a recurrent infection, the virus needs to evade its host’s immune responses for efficient viral propagation and pathogenicity.

In general, in virus-infected people, humoral immunity (mainly B cells) and cell-mediated immunity (mainly T cells, NK cells, and NKT cells) are induced at the time of the initial infection. Naïve CD8^+^ T cells recognize exogenous antigens via the major histocompatibility complex (MHC) class I complex and accelerate proliferation and differentiation into cytotoxic T lymphocytes (CTLs). CTLs contribute to efficient elimination of virus-infected cells. After eliminating the virus, CTLs change to memory CD8^+^ T cells and are maintained in the host to prevent reinfection. Unlike T cell, NK cells do not have receptors to recognize specific antigens. Instead, NK cells express various activating and inhibitory receptors that control their functions, such as cytotoxicity. Cellular stress, such as viral infection, can induce the expression of ligands that are recognized by NK cell activating receptors. In contrast, NK cells express multiple types of inhibitory receptors that recognize ligands like MHC class I molecules. NKT cells are T cells that express marker molecules of some T cells and NK cells. The NKT cell T cell receptor (TCR) recognizes glycolipids and phospholipids presented by CD1d molecules on antigen-presenting cells. 

Herpesvirus recurrence can occur in the presence of the innate, humoral, and cell-mediated immune responses. Herpesviruses acquired various strategies for host immune evasion, enabling them to efficiently establish recurrent infections. Several herpesvirus proteins and miRNAs that inhibit the innate immune response were identified and suggested to contribute to efficient viral propagation in vivo [7,8,9,10]. Therefore, in this review we focus on the mechanisms used by alphaherpesvirus viral proteins and miRNAs to evade the cell-mediated immune response, particularly those that evade the cell-mediated killing of infected cells.

## 2. Evasion of the Cell-Mediated Immune Response by Alphaherpesviruses

### 2.1. MHC Class I Antigen Presentation to CTLs

CTLs play a critical role in the control of herpesvirus infections in vivo [11,12]. Some of the viral proteins in virus-infected cells are degraded to peptides by cellular proteasomes. These viral peptides are translocated into the endoplasmic reticulum (ER) by the transporter associated with antigen processing (TAP) protein complex. MHC class I molecules on the ER membrane are associated with Tapasin, ERp57, and calreticulin to form an MHC class I peptide-loading complex that translocates viral peptides into the peptide-binding groove of MHC class I molecules. The MHC class I molecules are then released from the ER and transported to the cell surface via the Golgi apparatus for antigen presentation. CTLs recognize viral antigens on MHC class I molecules and release effectors, such as perforin, granzyme A and B, and interferon-γ (IFN-γ), to damage virus-infected cells, resulting in apoptosis [13]. Therefore, the MHC class I antigen presentation pathway seems to be a prime target for herpesvirus evasion of host immune surveillance [11,12]. In agreement with this possibility, several studies showed that herpesviruses evolved various strategies to inhibit MHC class I antigen presentation in cell cultures [11,12]. These in vitro mechanisms were reported to also affect viral propagation and pathogenicity in vivo in some animal betaherpesvirus and gammaherpesvirus infections [14,15,16].

#### 2.1.1. Inhibition of MHC Class I Antigen Presentation by HSV

HSV infection of human fibroblasts rapidly makes the cells resistant to lysis by HSV-specific CTLs, which normally recognize MHC class I proteins and viral peptides at the cell surface [17]. At least three HSV proteins inhibit the MHC class I antigen presentation pathway, suggesting that viral evasion of CTLs results in efficient viral propagation and pathogenicity in vivo. 

**Figure 1:**
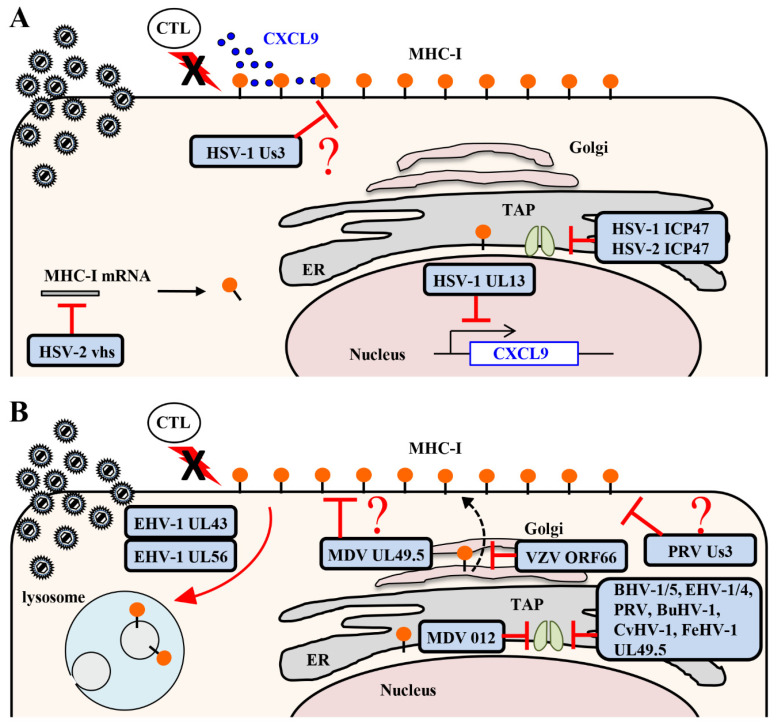
Alphaherpesviruses have multiple strategies to evade an infected cell’s cytotoxic T lymphocyte (CTL) response. (**A**) Herpes simplex virus 1 (HSV-1) Us3 mediates downregulation of major histocompatibility complex (MHC) class I surface expression. HSV ICP47 directly interacts with the transporter associated with antigen processing (TAP) and blocks binding of viral peptides to the TAP complex to inhibit viral antigen presentation on MHC class I molecules. HSV-2 virion host shutoff (vhs) degrades MHC class I mRNA, thereby reducing MHC class I protein expression. HSV-1 UL13 mediates downregulation of CXCL9 expression at infection sites to inhibit the accumulation of CTLs. (**B**) Varicella zoster virus (VZV) ORF66 mediates downregulation of MHC class I surface expression by retention of MHC class I molecules in the Golgi. pseudorabies virus (PRV) Us3 mediates downregulation of MHC class I surface expression. Varicellovirus (i.e., BHV-1/5, EHV-1/4, PRV, BuHV-1, CvHV-1 and FeHV-1) UL49.5 inhibits the function of TAP through a variety of mechanisms, resulting in downregulation of MHC class I molecules at the cell surface. EHV-1 UL56 mediates internalization and lysosomal degradation of MHC class I molecules cooperatively with EHV-1 UL43. Marek’s disease virus (MDV) UL49.5 mediates downregulation of MHC class I surface expression and MDV012 inhibits the TAP function.

ICP47 is a cytoplasmic membrane-associated protein that is encoded by the HSV Us12 gene [1]. ICP47 directly interacts with the TAP complex and blocks viral peptide binding to the TAP complex, thereby inhibiting viral antigen presentation by MHC class I molecules to CTLs in cell cultures (Figure 1A) [18,19]. Cryo-electron microscopy showed that HSV-1 ICP47 forms a long helical hairpin structure that blocks the TAP peptide translocation pathway from the cytoplasmic side [20,21]. The association of ICP47 with TAP interferes with substrate binding and prevents closure of the nucleotide binding domain necessary for ATP hydrolysis [20,21]. Although ICP47 binds tightly to human TAP, binding to murine TAP was reduced about 100-fold compared to human TAP [19,22]. Consistent with these data, ICP47 was shown to contribute to the protection of HSV-infected human fibroblasts from CTLs, but mouse fibroblasts were not protected [17]. The weak binding between ICP47 and murine TAP made it difficult to analyze the significance of ICP47 inhibition of MHC class I antigen presentation on viral pathogenicity in vivo. In contrast, despite the limited ability of ICP47 to inhibit murine TAP, a role for ICP47 in the evasion of CTL-mediated immunity in mice was suggested by a study showing that CTLs were able to protect mice from ICP47-deficient HSV-1 but not from wild-type HSV-1 [23]. Thus, the mechanism by which ICP47 acts in HSV evasion of CD8^+^ CTLs in mice remains uncertain at present. 

HSV-1 Us3 is a serine/threonine protein kinase that is conserved in alphaherpesviruses [1]. Us3 was reported to phosphorylate HSV-1 envelope glycoprotein B (gB) Thr-887 and cause downregulation of gB surface expression by promoting endocytosis of gB [24]. The Us3 kinase was also shown to mediate downregulation of MHC class I surface expression, although Us3 cannot directly phosphorylate MHC class I proteins in vitro and Us3 overexpression had no effect on MHC class I surface expression in cell cultures (Figure 1A). This suggested that Us3 indirectly downregulates MHC class I surface expression and that an additional viral and/or cellular component(s) may be required for Us3-mediated downregulation of MHC class I in HSV-1-infected cells [25]. Downregulation of MHC class I by Us3 protein kinase causes evasion of HSV-1-infected cells by HSV-1 gB_498–505_ epitope-specific CTLs in cell cultures [25]. As expected, more HSV-1-specific CD8^+^ T cells were induced in mice infected with a recombinant virus encoding a Us3 kinase-dead mutant than in mice infected with a recombinant virus in which the kinase-dead mutation was repaired [25]. Depletion of CD8^+^ T cells increased the replication of a Us3 kinase-dead mutant virus at the infection site following footpad infection in mice, compared to a mock depletion, indicating that Us3 protein kinase mediates evasion of HSV-1-specific CD8^+^ T cells by downregulating MHC class I surface expression and contributes to viral propagation in vivo [25]. MHC class I molecules also function as ligands for inhibitory receptors on NK cells [26]. Although Us3 protein kinase is required for the susceptibility of HSV-1-infected cells to NK cell recognition in vitro, depletion of NK cells had no effect on the replication of a Us3 kinase-dead mutant virus at the site of infection following footpad infection in mice [25]. Therefore, the effect of MHC class I downregulation by Us3 protein kinase on NK cells in vivo remains to be elucidated. Although the precise mechanism of Us3 protein kinase downregulation of MHC class I in HSV-1-infected cells remains unclear, Us3 protein kinase does inhibit the expansion of HSV-1-specific CD8^+^ T cells to enable efficient viral propagation in vivo [25].

The virion host shutoff (vhs) protein that is encoded by the HSV UL41 gene is a tegument protein with endoribonuclease activity and is conserved in alphaherpesviruses [1]. Although there is little specificity in RNA degradation by vhs in vitro, vhs preferentially degrades translating mRNAs in HSV-infected cells, resulting in a rapid shutoff of host cell protein synthesis (Figure 1A) [27,28]. The vhs protein also degrades MHC class I mRNA and inhibits synthesis of MHC class I proteins in HSV-infected cells [29]. The reduction of MHC class I proteins, mediated by vhs, was shown to enable HSV-2 infected cells to evade lysis by CTLs in cell cultures [29]. Therefore, it would be expected that propagation and pathogenicity of the vhs-deficient HSV-2 increased in the absence of T cells in vivo compared to that in the presence of T cells. However, viral propagation and pathogenicity in vhs-deficient, HSV-2-infected SCID mice that lacked functional B and T cells were reduced to levels similar to those in wild-type mice [30]. These results indicate that vhs apparently is not involved in virus evasion of CTLs in vivo. Therefore, it is important to determine whether evasion of CTLs by HSV viral proteins in HSV-infected cells in vitro is also significant in viral propagation and pathogenicity in vivo.

#### 2.1.2. Inhibition of MHC Class I Antigen Presentation by Varicelloviruses

Like other alphaherpesviruses, VZV, an alphaherpesvirus in the genus Varicellovirus, also reduces MHC class I surface expression in infected human fibroblasts [31]. VZV ORF66 is a serine/threonine protein kinase, a homolog of HSV-1 Us3, and mediates downregulation of MHC class I surface expression in VZV-infected cells (Figure 1B) [31]. Although it is not known whether ORF66 directly phosphorylates MHC class I proteins, overexpression of ORF66 reduces MHC class I surface expression in cell cultures in a kinase activity dependent manner, suggesting that ORF66 can mediate downregulation of MHC class I surface expression independent of other viral proteins [31,32]. VZV infection downregulates MHC class I surface expression by impairing the transport of MHC class I molecules from the Golgi compartment to the cell surface. Pulse-chase studies combined with endo H cleavage analysis showed that ORF66 induces the retention of MHC class I molecules in the Golgi [32]. It is unclear how suppression of MHC class I surface expression by ORF66 affects efficient viral propagation and pathogenicity in vivo since, unlike HSV, VZV has a limited host range in animal models (e.g., mice cannot be easily infected).

PRV Us3 is a serine/threonine protein kinase that is a homolog of HSV-1 Us3 and mediates downregulation of MHC class I surface expression in PRV-infected cells (Figure 1B) [33]. Interestingly, PRV Us3 is necessary for downregulation of MHC class I surface expression in ST cells derived from porcine fetal testes, but not in porcine kidney (PK-15) cells or in porcine alveolar macrophages (PAM) [33]. Overexpression of Us3 was shown to have no effect on MHC class I surface expression in ST cells, suggesting that an additional viral and/or cellular component(s) may be involved in Us3-mediated downregulation of MHC class I in PRV-infected cells [33].

UL49.5, also designated glycoprotein N (gN), in alphaherpesviruses is a type I transmembrane protein and interacts with glycoprotein M (gM). gM and UL49.5 are conserved in all herpesviruses and are involved in viral penetration and egress [34]. UL49.5 in varicelloviruses (i.e., BHV-1, EHV-1/4 and PRV) inhibits the function of TAP by several different mechanisms, resulting in downregulation of MHC class I molecules at the cell surface (Figure 1B) [33,35,36,37,38], as follows. (1) BHV-1 UL49.5 interacts with the MHC class I peptide-loading complex to inhibit peptide translocation by TAP in infected cells [35,36]. MHC class I surface expression in cells expressing BHV-1 UL49.5 is reduced compared to that in control cells [35,36]. In these BHV-1 UL49.5-expressing cells, TAP is degraded by the proteasome; the cytoplasmic tail of UL49.5 is essential for the degradation of TAP, but not for inhibition of peptide translocation [35]. BHV-1 UL49.5 does not inhibit TAP binding to ATP or peptides, but does inhibit the conformational change in TAP associated with peptide translocation and MHC class I-restricted T cell recognition [35,36]. Like BHV-1 UL49.5, BHV-5 UL49.5 mediates degradation of TAP, inhibits peptide translocation by TAP, and reduces MHC class I surface expression in UL49.5-expressing cells [39]. (2) MHC class I surface expression in EHV-1 UL49.5-expressing cells is reduced compared to that in control cells [36]. In EHV-1 UL49.5-expressing cells, UL49.5 does not affect TAP steady state levels or peptide binding to TAP, but does inhibit TAP binding to ATP and TAP conformational change [36]. UL49.5 also inhibits ATP binding to TAP in EHV-4 UL49.5-expressing cells and peptide translocation by TAP in EHV-1 and EHV-4 infected cells [36,38]. However, UL49.5 has no effect on downregulation of MHC class I surface expression in EHV-1 and EHV-4 infected cells; the level of MHC class I molecules at the surface of cells infected with the UL49.5-deficient virus is similar to that in cells infected with wild-type virus [38,40]. These results may be cell-type dependent since they were determined in different cell types. In addition, it is unclear how inhibition of the TAP function by UL49.5 in EHV-1-infected cells affects CTL recognition, so further analysis is needed. (3) PRV UL49.5 also mediates downregulation of MHC class I surface expression in a cell-type dependent manner. PRV UL49.5 mediates downregulation of MHC class I surface expression and is responsible for the inhibition of peptide translocation by TAP in PRV-infected PK-15 cells [33,35], but PRV UL49.5 has no effect on downregulation of MHC class I surface expression in PRV-infected ST cells and PAM cells [33]. MHC class I surface expression in UL49.5-expressing PK15 cells is reduced compared to that in control cells [36]. In PRV UL49.5-expressing MJS cells, UL49.5 does not affect TAP steady state levels or TAP binding to peptides and ATP, but does inhibit the TAP conformational change [36]. These observations suggest that PRV UL49.5 is necessary and sufficient for downregulation of MHC class I surface expression in a cell-type dependent manner. (4) In other varicelloviruses, such as bubaline herpesvirus 1 (BuHV-1) and cervid herpesvirus 1 (CvHV-1), UL49.5 also mediates degradation of TAP, inhibits peptide translocation by TAP, and reduces MHC class I surface expression [37]. Felid herpesvirus 1 (FeHV-1) UL49.5 also inhibits peptide translocation by TAP and reduces MHC class I surface expression, but does not mediate TAP degradation [37]. Although VZV UL49.5 interacts with the peptide-loading complex, it does not block TAP function, reduce MHC class I surface expression, or inhibit MHC class I-restricted T cell recognition [36]. HSV-2 UL49.5 also has no effect on MHC class I surface expression [35]. Although UL49.5 has low amino acid sequence homology in varicelloviruses, the mechanism of TAP inhibition by UL49.5 is conserved in many varicelloviruses, suggesting that a specific amino acid sequence or structure may be important. The impact of TAP inhibition by varicellovirus UL49.5 on viral propagation and pathogenicity in vivo remains to be elucidated. 

UL56 in EHV-1 and EHV-4 is encoded by the ORF1 gene, which is a homolog of HSV-1 UL56 and is predominantly localized in the Golgi complex in infected cells [40]. UL56 in EHV-1 and EHV-4 mediates downregulation of MHC class I surface expression in EHV-1-infected cells (Figure 1B) [38,40]. EHV-4 UL56 has no effect on peptide translocation by TAP in infected cells [38]. Cell surface MHC class I molecules are internalized and degraded in the lysosomal compartment of EHV-1 infected cells in a UL56-dependent manner [41]. Endocytosis of cell surface MHC class I molecules mediated by UL56 is dependent on dynamin, but not on clathrin or caveolin-1 [41]. Studies with PYR41, the inhibitor of ubiquitin-activating enzyme E1, suggested that downregulation of MHC class I surface expression caused by EHV-1 infection requires ubiquitination [41]. UL56 overexpression has no effect on MHC class I surface expression in cell cultures, suggesting that an additional viral and/or cellular component(s) may be required for UL56-mediated downregulation of MHC class I in EHV-infected cells [38,40]. EHV-1 UL43 is encoded by the ORF17 gene, localized in Golgi vesicles, and mediates downregulation of MHC class I surface expression in EHV-1-infected cells (Figure 1B) [42]. However, UL43 overexpression, like UL56 overexpression, has no effect on MHC class I surface expression in cell cultures on its own, but co-expression of UL56 and UL43 reduces MHC class I surface expression [42]. UL43 is an early gene product and is degraded in lysosomes [42]. Like the UL43 homolog in HSV-1 and PRV, EHV-1 UL43 is a putative type III transmembrane protein, with the hydrophilic domain at its N terminus essential for downregulation of MHC class I surface expression in infected cells [42]. 

#### 2.1.3. Inhibition of MHC Class I Antigen Presentation by Mardiviruses

MDV infection, like other alphaherpesvirus infections, reduces MHC class I surface expression in chicken fibroblasts [43,44]. Like some varicellovirus UL49.5 proteins, MHC class I surface expression in MDV UL49.5-expressing cells is reduced compared to that in control cells [45]. In addition, downregulation of MHC class I surface expression was shown to be greatly reduced in cells infected with an MDV UL49.5 cytoplasmic tail-deficient mutant virus, suggesting that MDV UL49.5 mediates downregulation of MHC class I surface expression in infected cells (Figure 1B) [45]. In contrast, other studies reported that MHC class I surface expression in MDV UL49.5-expressing cells was similar to that in control cells [37]. These results may be cell-type dependent since they were determined in different cell types. Therefore, further detailed analysis is needed to evaluate the effect of MDV UL49.5 on MHC class I surface expression.

MDV012 is a 489 amino acid protein encoded by two exons separated by an 83 bp intron and is found in some mardiviruses, i.e., Gallid Herpesvirus 3 (GaHV-3), Duck Enteritis Virus (DEV), and Meleagrid herpesvirus 1 (MeHV-1). MHC class I surface expression in MDV012-expressing cells is downregulated compared to that in control cells (Figure 1B) [46]. It is unclear whether MDV012 targets TAP and inhibits antigen presentation by MHC class I in MDV-infected cells and, as a result, contributes to efficient viral propagation and pathogenicity in vivo. 

### 2.2. Infiltration of CTLs at Infection Sites by Chemokines

Chemokines are cytokines that induce cell migration activity and control intratissue movement and localization of various immune cells [47]. It is well-known that chemokines are involved in the development of various diseases, such as inflammatory diseases, autoimmune diseases, and various infectious diseases [47]. All chemokines activate seven transmembrane receptors coupled to heterotrimeric G proteins to transmit signals [47]. Leukocytes express various chemokine receptors and are able to selectively and efficiently invade inflamed and/or virus-infected tissues [47]. In addition, leukocytes regulate their functions by modulating the expression of chemokine receptors on their cell surface in response to various stimuli [47]. Chemokines not only induce cell chemotaxis by leukocytes, but also activate the leukocytes themselves and promote the release of mediators [47]. In herpesvirus infections, various chemokines are produced at infection sites, promote infiltration by leukocytes and, as a result, contribute to regulating viral propagation and pathogenicity [48,49,50]. When mice were infected with HSV, chemokine (C-X-C motif) ligand 9-11 (CXCL9-11) expression was induced at the infection sites [51,52]. These chemokines attract T cells and NK cells that express the chemokine receptor C-X-C Motif Chemokine Receptor 3 (CXCR3), which uses the chemokines as ligands to infiltrate infected sites [51,53]. These cytotoxic lymphocytes damage HSV-infected cells and contribute to eliminating infected cells. Therefore, the inhibition of expression of these chemokines by HSV inhibits the infiltration of CTLs and NK cells into HSV infection sites, resulting in efficient viral propagation and pathogenicity. In agreement with this notion, herpesviruses encode various homologs of chemokines or their receptors, which appear to be important for immune evasion of these viruses [54,55]. However, until recently, it was unclear whether HSV suppresses invasion of immune cells by inhibiting the expression of these chemokines or by inhibiting their activity.

#### Downregulation of Chemokine CXCL9 Expression by HSV-1 

HSV-1 UL13 is a serine/threonine protein kinase that is conserved in all herpesviruses [1]. It was suggested that HSV-1 UL13 promotes viral propagation, expression of a subset of viral genes in cell cultures in a cell-type dependent manner, and may mimic a cellular cyclin-dependent protein kinase [56,57,58]. Following ocular infection, less mice infected with a recombinant virus encoding a UL13 kinase-dead mutant died than mice infected with a recombinant virus in which the UL13 kinase-dead mutation were healed [59]. Interestingly, although UL13 kinase activity has no effect on viral propagation and antigen spread in the brains of mice following ocular infection, it is required for evasion of the elimination of HSV-1-infected cells in the brains of infected mice [59]. In addition, HSV-1 UL13 was shown to mediate downregulation of CXCL9 expression in the brains of mice following ocular infection [59]. In ocular infections of mice with a recombinant virus encoding a UL13 kinase-dead mutant, more HSV-1-specific CD8^+^ T cells were induced than in mice infected with a recombinant virus in which the UL13 kinase-dead mutation was repaired [59]. Depletion of CD8^+^ T cells inhibited elimination of UL13 kinase-dead mutant viruses at the site of infection and the mice died, indicating that UL13 protein kinase mediates viral evasion of HSV-1-specific CD8^+^ T cells by downregulating CXCL9 expression in the infected mouse brains, resulting in efficient viral propagation and pathogenicity in vivo (Figure 1A) [59]. Interestingly, administration of CXCL9 in the infection site in mouse brains infected with a recombinant virus in which the UL13 kinase-dead mutation was repaired increased the accumulation of HSV-1-specific CD8^+^ T cells and inhibited efficient viral propagation and pathogenicity [59]. Therefore, it may be a useful therapy against HSV encephalitis if CXCL9 could be expressed safely at an HSV infection site in the brain, possibly by a viral vector rather than direct administration. In addition, the UL13 kinase-dead mutant virus had no effect on the mortality of infected CXCL9-deficient mice. However, the UL13 kinase-dead repaired mutant virus produced less mortality in infected CXCL9-deficient mice than in wild-type mice, suggesting that CXCL9 expression is also required for the mortality of mice following ocular infection. Although the precise mechanism of UL13 protein kinase downregulation of CXCL9 expression in the brains of mice following ocular infection is not known, UL13 protein kinase does inhibit the accumulation of HSV-1-specific CD8^+^ T cells at the infection site, thereby enabling efficient viral propagation and pathogenicity in vivo [59]. 

### 2.3. Natural Killer Cells

Natural killer (NK) cells are lymphocytes that directly recognize virus-infected cells and tumor cells, and have cytotoxic activity [60,61]. Unlike T cells and B cells, NK cells do not have receptors that recognize specific antigens [26]. NK cells have multiple activating and inhibitory receptors and bind to activating and suppressing ligands expressed in virus-infected cells and tumor cells; their cytotoxic activity against target cells is controlled by the expression of these ligands [62]. In particular, to directly recognize virus-infected cells and tumor cells, NK cells have killer cell immunoglobulin-like receptors (KIRs) that are inhibitory receptors and recognize MHC class I as a suppressing ligand [26]. MHC class I molecules are expressed on the cell surface of normal cells and are recognized by the KIRs on NK cells, so normal cells are not damaged [26]. However, in virus-infected cells and some tumor cells, MHC class I surface expression is downregulated and the recognition ability of MHC class I by KIRs on NK cells is decreased. Therefore, NK cells react to these cells as “missing-self” and damage them [60,61]. 

Downregulation of MHC class I surface expression contributes to viral evasion of damage by cytotoxic T cells, but increases their susceptibility to damage by NK cells [26]. Therefore, downregulation of MHC class I surface expression has both positive and negative effects on viruses. Some viruses acquired a mechanism to downregulate the surface expression of NK cell activating ligands to avoid damage by NK cells. Expression of CD112 (nectin-2) and CD155 (poliovirus receptor; PVR), which are NK cell activating ligands, is increased in virus-infected cells and tumor cells. NK cells recognize CD112 and CD155 by the DNAX accessory molecule 1 (DNAM-1), one of the activating receptors expressed on the cell surface, and damage virus-infected cells and tumor cells [26]. The natural killer cell group 2, member D (NKG2D) receptor is also one of the activating receptors expressed on NK cells and CD8^+^ T cells. NKG2D ligands in human cells are MHC class I polypeptide-related sequence A (MICA) and B (MICB) proteins and UL16-binding proteins (ULBP)-1–6; these ligands are not expressed or have lower expression in normal cells [26]. However, surface expression of the NKG2D ligand is enhanced in virus-infected cells and tumor cells. Therefore, the NKG2D receptor on NK cells recognizes these ligands and the activated NK cells damage virus-infected cells and tumor cells [60,61]. Among other receptors expressed on the surface of NK cells, 2B4 is one of the NK cell activating receptors and recognizes CD48 as a ligand [26]. As described above, MHC class I surface expression is decreased in alphaherpesvirus-infected cells. Therefore, alphaherpesvirus-infected cells can be a target of NK cells, but several alphaherpesviruses avoid damage from NK cells by suppressing the surface expression of NK cell activating ligands or upregulating the surface expression of NK cell inhibitory ligands. NK cell activating and inhibitory ligands are species-specific molecules and the corresponding ligands are also expressed in mice. Since the effect of NK cell evasion on alphaherpesvirus propagation and pathogenicity in vivo cannot always be analyzed in mice, the significance of NK cell evasion by HSV in vivo remains unclear.

#### 2.3.1. Downregulation of the Cell Surface Expression of Activating Ligands by HSV

**Figure 2:**
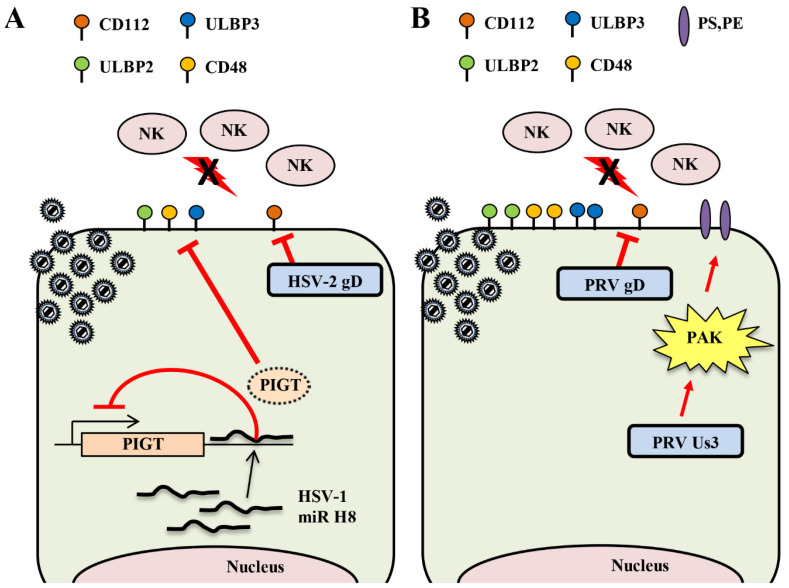
Alphaherpesviruses have multiple ways to evade the NK cell response. (**A**) HSV-2 gD mediates downregulation of CD112 surface expression. HSV-1 miR-H8 inhibits the expression of PIGT, which is one of the factors forming the GPI transamidase complex, thereby reducing cell surface expression of ULBP2, 3 and CD48. (**B**) PRV gD mediates downregulation of CD112 cell surface expression. PRV Us3 activates Group 1 p21-activated kinases (PAKs), resulting in upregulation of PS surface expression, which binds the CD300a inhibitory NK cell receptor.

CD112 is an activating ligand for DNAM-1 and, in HSV-2 infections, CD112 is a gD receptor [63,64]. However, HSV-1 gD does not use CD112 as an entry receptor, except for strains isolated from some HSV-1 encephalitis patients [65]. HSV-1 gD uses the nectin family CD111 (nectin-1) as an entry receptor [63]; HSV-1 gD can suppress nectin-1 surface expression by transinteraction [66]. Therefore, the effect of HSV-2 gD on CD112 surface expression in HSV-2 infected cells was analyzed. Similar to the downregulation of CD111 surface expression by HSV-1 gD, the cell surface level of CD112 was also decreased in HSV-2 infected cells compared to uninfected cells. However, the cell surface level of CD112 in cells infected with an HSV-2 gD-deficient virus was comparable to that in uninfected cells [67]. Therefore, in addition to using CD112 as an entry receptor, HSV-2 gD suppresses CD112 surface expression in HSV-2 infected cells to avoid damage by NK cells (Figure 2A). The effect of NK cell evasion by HSV-2 gD on viral propagation and pathogenicity in vivo is not known and further analysis is needed.

HSV-1 and HSV-2 encode at least 27 and 24 different miRNA sequences, respectively, and these miRNAs are expressed during the virus infection cycle, but little is known about their function [9]. It was reported that miRNA encoded by human cytomegalovirus (HCMV) suppresses the surface expression of MICB, which is one of the NK cell activating ligands, and thereby contributes to the evasion of damage by NK cells [68]. It was shown that miR-H8, which is an HSV miRNA with a sequence homologous to the 3 ‘UTR of the PIGT gene, similarly suppresses expression of the NK activating ligand [69]. PIGT is one of the components of the GPI transamidase complex [70]. Exogenous expression of miR-H8 suppresses the surface expression of GPI anchor proteins (i.e., ULBP2, ULBP3, and CD48) by suppressing PIGT expression (Figure 2A). The exogenous expression of miR-H8 also decreases cytotoxicity by NK cells [69]. However, the effect of miR-H8 on surface expression of the ULBP2, ULBP3, and CD48 gene products in HSV-1-infected cells is not known. Further studies are needed using recombinant HSV-1 that does not express miR-H8 to clarify the significance of miR-H8 in HSV-1-infected cells. 

#### 2.3.2. Downregulation of the Surface Expression of NK Activating Ligands by Varicelloviruses

PRV and BHV-1 gD also interact with CD112 as an entry receptor [63,64]. In wild-type, PRV-infected cells, the cell surface level of CD112 is reduced compared to uninfected cells, but in gD-deficient, PRV-infected cells, the cell surface level of CD112 is similar to that of uninfected cells (Figure 2B) [67]. In addition, transient expression of PRV gD significantly reduces the cell surface level of CD112 [67]. In agreement with this, NK cell cytotoxic activity mediated by DNAM-1, which is the NK cell activating receptor for the CD112 ligand, is more efficient in gD-deficient, PRV-infected cells than in wild-type, PRV-infected cells [67]. In addition, DNAM-1 mediated NK cell cytotoxic activity is significantly reduced even with transient expression of PRV gD [67]. The amount of CD112 protein is apparently reduced in wild-type, PRV-infected cells compared to uninfected cells, but the amount of CD112 protein in gD-deficient, PRV-infected cells is similar to that in uninfected cells [67]. PRV gD degrades CD112 in an acidification-dependent manner, since the addition of an acidification inhibitor to wild-type, PRV-infected cells suppresses the proteolysis of CD112 [67]. However, it is unclear how the degradation of CD112 by PRV gD contributes to the evasion of NK cell damage in vivo and to PRV replication and pathogenicity.

#### 2.3.3. Upregulation of the Surface Expression of NK Inhibitory Ligands by Varicelloviruses

CD300a is an inhibitory NK cell receptor in the immunoglobulin (Ig) IRp60 superfamily and possesses phosphatidylserine (PS) and phosphatidylethanolamine (PE) phospholipids as ligands [71,72]. Wild-type, PRV-infected cells exhibit enhanced binding to CD300a, but Us3-deficient, PRV-infected cells do not exhibit enhanced binding to CD300a [73]. Therefore, in PRV-infected cells, CD300a binding is enhanced in a Us3-dependent manner; the level of surface expression of PS in PRV-infected cells is maintained at the same level as in uninfected cells, but the level of surface expression of PS in Us3-deficient PRV-infected cells and Us3 kinase-dead PRV-infected cells is apparently reduced (Figure 2B) [73]. It was shown that activation of Group I p21-activated kinases (PAKs) in host cells contributes to the regulation of PS surface expression [74]. In addition, PRV Us3 phosphorylates and activates PAKs [75]. A study using IPA-3, which is an inhibitor of Group I PAKs, showed that upregulation of PS surface expression and enhancement of CD300a binding in PRV-infected cells were dependent on the Us3 PK activity and the Group I PAKs signaling pathway [73]. It remains unclear to what extent PAKs affect viral propagation and pathogenicity in vivo.

### 2.4. Natural Killer T Cells

Natural killer T (NKT) cells express marker molecules of T cells (i.e., T-cell receptors, TCRs) and NK cells [76]. NKT cells produce various cytokines, including IFN-γ, that mediate activation of cells, such as T cells, NK cells, and B cells, and contribute to the regulation of viral infections [76]. NKT cell TCRs recognize glycolipids, such as α-galactosylceramide (αGalCer, a glycolipid antigen derived from marine sponges and pathogen-derived lipids) and phospholipids presented by CD1d molecules on antigen-presenting cells [76]. As with MHC class I molecules, CD1d molecules are β2-microglobulin-associated MHC-like molecules, but are not diverse like MHC class I molecules [76]. Nevertheless, CD1d binds to a number of glycolipids and can present various antigens to NKT cells. Bacterial and fungal glycolipids are also ligands that activate NKT cells [76]. There are no similar viral glycolipids, suggesting that host-derived self-antigens contribute to the activation of NKT cells via pattern recognition receptors (PRRs), such as the Toll-like receptors (TLRs), during viral infection. 

Regarding the significance of NKT cells in HSV infections, it was suggested that NKT cells are responsible for host defense and are important for HSV infection control, since viral propagation and pathogenicity increase in CD1d-deficient mice infected with HSV-1 compared to infection in wild-type mice [77,78]. However, some other studies reported that NKT cells are not important for anti-HSV-1 immunity [79]. These different results may be due to the HSV-1 strains and/or mouse strains used in these in vivo experiments. Further analysis is needed on the significance of NKT cells in HSV-1 infection control. Nevertheless, the CD1d antigen-presentation pathway seems to be a prime target for herpesviruses to evade a host immune response by NKT cells. 

#### Inhibition of CD1d Recycling by HSV-1 Us3 and gB

**Figure 3:**
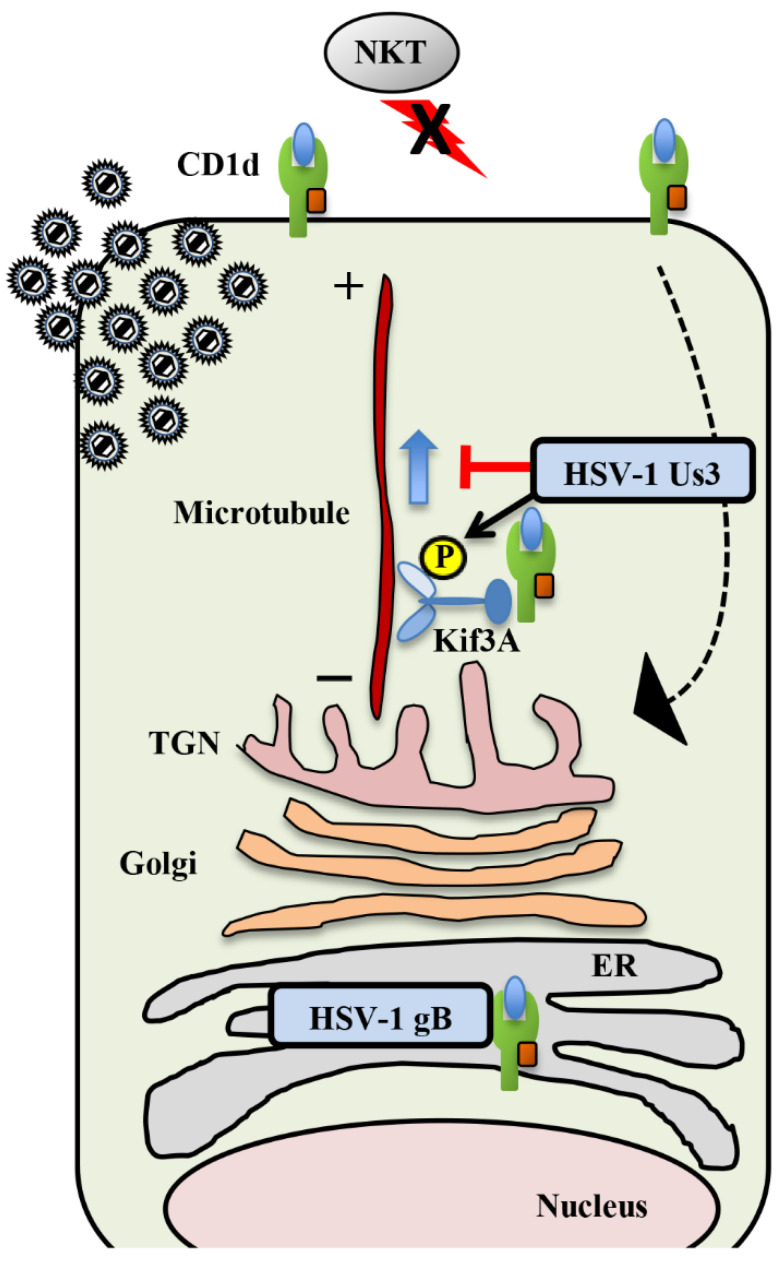
HSV-1 inhibits CD1d-restricted antigen presentation to natural killer T (NKT) cells. HSV-1 gB and Us3 mediate inhibition of CD1d recycling to suppress CD1d surface expression. HSV-1 gB interacts with CD1d, but this is not sufficient to inhibit CD1d surface expression. Us3 phosphorylates KIF3A Ser-687, which is required for suppression of CD1d surface expression.

The surface expression of CD1d is downregulated in HSV-infected cells [80]. As a result, HSV-infected cells evade damage mediated by NKT cells [80]. The CD1d protein level in HSV-1-infected cells is similar to that in uninfected cells, indicating that downregulation of CD1d surface expression is not caused by suppression of the amount of CD1d by HSV-1 infection [80]. The vhs protein is an HSV endoribonuclease and does not affect the downregulation of CD1d surface expression in infected cells [80]. Downregulation of CD1d surface expression in HSV-1-infected cells is due to the inhibition of CD1d recycling to the cell surface [80]. In a study of the inhibitory mechanism of CD1d recycling, gB was identified as the viral factor that interacts with CD1d (Figure 3) [81]. The surface expression of CD1d in cells infected with gB-deficient HSV-1 was not downregulated compared to that in wild-type, HSV-1-infected cells. Even if gB is transiently transfected into the cells, the surface expression of CD1d was similar to that of cells that were not transfected, suggesting that gB is necessary but not sufficient for inhibiting CD1d recycling [81]. A previous study showed that gB is phosphorylated by Us3 and that this phosphorylation regulates the surface expression of gB [24]. When gB and Us3 were transiently co-transfected into cells, the surface expression of CD1d was suppressed [81]. In addition, it was suggested that Us3 regulates the co-localization of gB and CD1d, thereby controlling the cell surface level of CD1d (Figure 3) [81]. Both gB and Us3 are required for the inhibition of CD1d-restricted NKT cell activation in HSV-1 infected cells [81]. Further analysis identified KIF3A as a host factor contributing to the suppression of CD1d surface expression via Us3 [82]. KIF3A is also an important motor protein for CD1d surface expression, since knockdown of KIF3A expression suppresses the surface expression of CD1d in mock-infected cells [82]. Us3 interacts with KIF3A and phosphorylates KIF3A Ser-687 (Figure 3) [82]. Overexpression of Us3 and wild-type KIF3A in HeLa cells decreases CD1d surface expression compared to overexpression of Us3 and the KIF3A S687A mutant, suggesting that phosphorylation of KIF3A Ser-687 is required for efficient downregulation of CD1d surface expression by Us3 [82]. Interestingly, mouse CD1d surface expression was not downregulated by HSV-1 infection, suggesting that HSV-1 evolved to specifically evade human CD1d and NKT cells for efficient pathogenicity [83]. As expected, after ocular infection of CD1d-humanized mice, wild-type HSV-1 replicated much more efficiently in mouse eyes than Us3-deficient HSV-1 [83]. In addition, although CD1d-humanized mice infected with wild-type HSV-1 showed periocular skin disease, Us3-deficient HSV-1 infected mice showed few symptoms [83]. Us3-deficient HSV-1 replicated much more efficiently in CD1d-deficient mice than in CD1d-humanized mice [83]. These observations indicate that Us3 mediates evasion of NKT cells by downregulating CD1d surface expression, which contributes to viral propagation and pathogenicity in vivo.

## 3. Conclusions and Future Perspectives

Herpesviruses establish life-long latent infections and recurrent lytic infections in humans and animals. To carry out these infections, herpesviruses acquired methods to evade the natural and adaptive immune antiviral responses of their hosts. This review outlines the methods alphaherpesviruses evolved to evade these adaptive antiviral responses. A number of alphaherpesvirus immune evasion strategies were studied previously, including suppression of MHC class I-restricted antigen presentation, suppression of CTL-induced chemokine expression, suppression of cell surface expression of NK cell activating ligands, induction of surface expression of NK cell inhibitory ligands, and CD1d suppression of antigen presentation to NKT cells. Since most of these studies were in cell cultures, it is important to assess the effect of viral immune evasion strategies on viral propagation and pathogenicity in vivo using animal models. 

As reviewed here, previous studies elucidated multiple mechanisms of alphaherpesvirus evasion of cell-mediated immunity. However, other currently unknown alphaherpesvirus immune evasion mechanisms remain to be identified. For example, multiple mechanisms to evade CTLs were identified in HSV-infected cells. However, little is known about the mechanism for HSV evasion of NK cells. There are multiple types of NK cell activation ligands, and it was shown that their cell surface levels in HSV-infected cells were reduced compared to those in uninfected cells, but the details of this reduction remain unknown [84]. Therefore, the elimination of HSV-infected cells by NK cells and their evasion by HSV affect viral propagation and pathogenicity in vivo remain unclear. These are very interesting questions of clinical importance, so future progress in their analysis should be expected.
